# A case of endoscopic therapy using electrohydraulic lithotripsy under peroral pancreatoscopy via the minor papilla

**DOI:** 10.1055/a-2791-4716

**Published:** 2026-02-17

**Authors:** Katsuyuki Dainaka, Takashi Otsuka, Juichiro Yoshida, Hideki Fujii, Naoya Tomatsuri, Yusuke Okuyama, Hideki Sato

**Affiliations:** 1 26335 Department of Gastroenterology, Kyoto First Red Cross Hospital, Kyoto, Japan

Recently, peroral pancreatoscopy (POPS) and electrohydraulic lithotripsy (EHL) have been identified as useful endoscopic treatment options for refractory pancreatic calculi 1 . However, owing to the technical challenges, POPS–EHL via the minor papilla has rarely been described. We report a case of refractory pancreatic calculi associated with incomplete pancreatic divisum successfully treated using POPS (eyeMAX 9 Fr; MICRO-TECH, Nanjing, China) and EHL via the minor papilla.

A 66-year-old man with idiopathic chronic pancreatitis presented with obstructive pancreatitis caused by pancreatic body calculi refractory to conservative therapy ( Fig. 1 ). Endoscopic retrograde cholangiopancreatography (ERCP) via the major papilla opacified the dorsal duct; however, neither a guidewire nor an endoscopic pancreatic stent (EPS) could initially traverse the suspected fusion segment. An EPS was placed proximally for post-ERCP pancreatitis prophylaxis. Because the stent could not cross the suspected fusion area, magnetic resonance cholangiopancreatography was subsequently performed and, in combination with ERCP findings, confirmed incomplete pancreatic divisum.

**Fig. 1:**
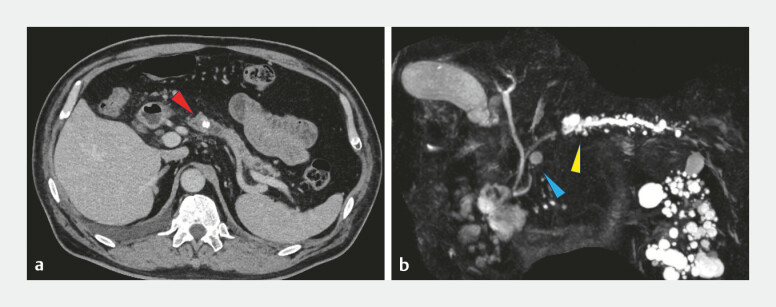
**a** Computed tomography of the main pancreatic duct calculus (body of the pancreas; red arrow). **b** Magnetic resonance cholangiopancreatography showed main pancreatic duct dilatation, distal to the calculus and lack of continuity between the ventral and dorsal pancreatic ducts, consistent with incomplete pancreatic divisum(yellow arrow).

Minor papilla cannulation enabled access to the Santorini duct, and pancreatography demonstrated an impacted pancreatic calculus causing ductal obstruction in the dominant dorsal duct. Although a guidewire could be advanced to the ductal segment at the level of the stone, the pancreatic stent could not be passed across the obstructed segment even after endoscopic minor papilla sphincterotomy, and it was therefore placed proximally ( Fig. 2 ). Extracorporeal shockwave lithotripsy was attempted but failed, after which stepwise ductal preparation was performed.

**Fig. 2:**
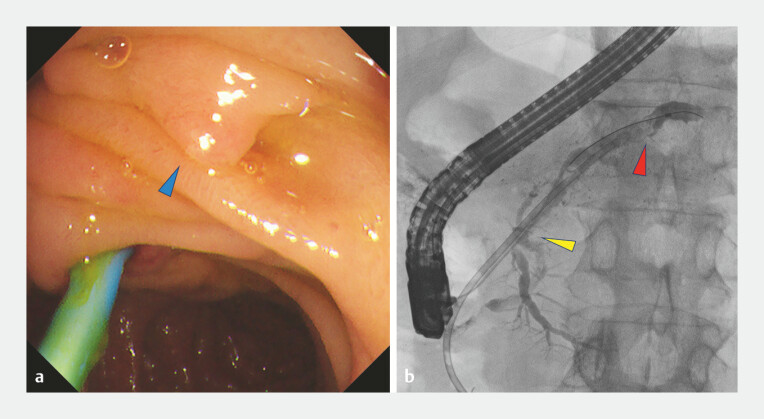
**a** Duodenoscopy showed minor papilla, cranial to major papilla containing the pancreatic duct stent (blue arrow). **b** Pancreatography of the dorsal (Santorini) duct demonstrating simultaneous opacification of the ventral duct as well (yellow arrow), supporting the diagnosis of incomplete pancreatic divisum. The endoscopic pancreatic stent could not be advanced beyond the level of the impacted stone and was therefore placed proximally, just before the obstruction site (red arrow).

Following balloon dilation, POPS was smoothly advanced through the Santorini duct ( Fig. 3 ), allowing the direct visualization of a white pancreatic calculus, which was successfully fragmented using the EHL ( Video 1 ). Adequate lithotripsy and POPS passage were confirmed, and a prophylactic EPS was placed. No complications occurred ( Fig. 4 ).

**Fig. 3:**
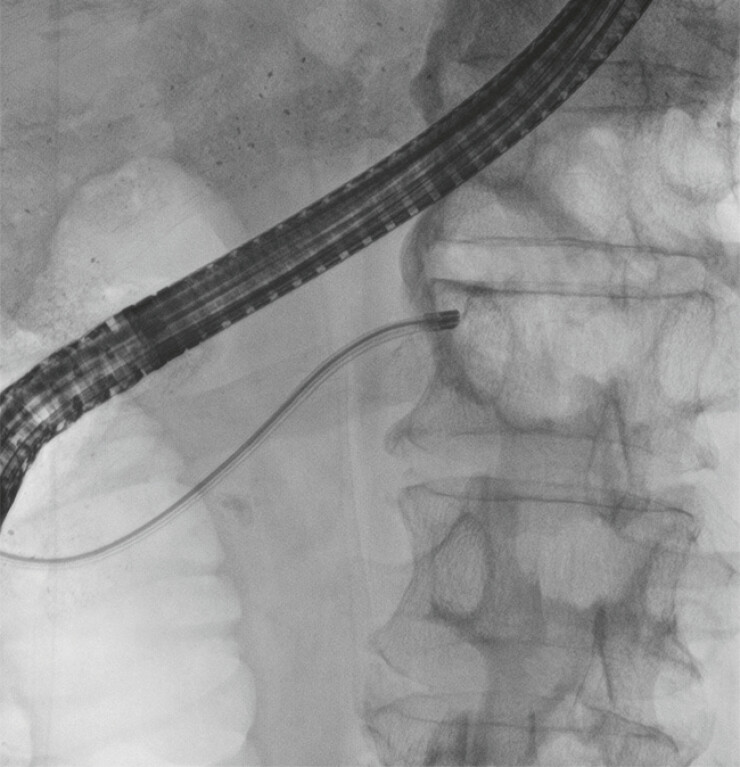
Radiographic images of POPS being advanced toward the pancreatic duct obstruction via the minor papilla. POPS, peroral pancreatoscopy.

Peroral pancreatoscopy via the minor papilla and electrohydraulic lithotripsy demonstrating stepwise ductal preparation, the minor papilla approach, and direct visualization with successful stone fragmentation.Video 1

**Fig. 4:**
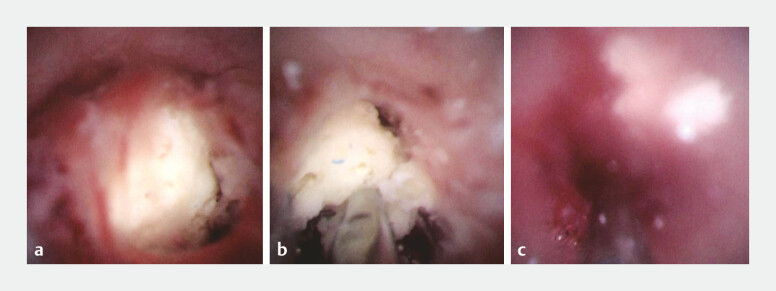
Peroral pancreatoscopy-based images. **a** Direct confirmation of pancreatic calculi. **b** Electrohydraulic lithotripsy fragmented the calculus. **c** The complete fragmentation of the pancreatic calculus enabled scope insertion across the previously obstructed ductal segment.

Although POPS occasionally causes severe complications 2 , careful anatomical assessment, and stepwise ductal preparation may help enhance procedural safety when performing POPS–EHL.

Endoscopy_UCTN_Code_CCL_1AZ_2AG Endoscopy_UCTN_Code_TTT_1AR_2AL

## References

[1] Han S Miley A Akshintala V Per-oral pancreatoscopy-guided lithotripsy vs. extracorporeal shock wave lithotripsy for treating refractory main pancreatic duct stones in chronic pancreatitis: Protocol for an open-label multi-center randomized clinical trial Pancreatology 2022 22 1120 1125 10.1016/j.pan.2022.09.245 36273991

[2] Huang P Khizar H Song W Pancreatoscopy-Guided Lithotripsy for Pancreatic Duct Stones: A Systematic Review and Meta-Analysis Turk J Gastroenterol 2024 35 811 821 10.5152/tjg.2024.24110 39548977 PMC11562744

